# Racial Biases Associated With Pulse Oximetry: Longitudinal Social Network Analysis of Social Media Advocacy Impact

**DOI:** 10.2196/56034

**Published:** 2024-10-08

**Authors:** Wasim Ahmed, Mariann Hardey, Bradford David Winters, Aarti Sarwal

**Affiliations:** 1 Marketing Management and Business Strategy Hull University Business School University of Hull Kingston Upon Hull United Kingdom; 2 Durham University Business School Durham University Durham United Kingdom; 3 Anesthesiology and Critical Care John Hopkins School of Medicine Baltimore, MD United States; 4 Neurology Atrium Wake Forest School of Medicine Winston-Salem, NC United States

**Keywords:** social media, X, racial biases, pulse oximetry, advocacy, impact, awareness, racial, bias, biases, longitudinal study, information, dissemination, disparity, disparities, accuracy, social network analysis, Academic Track application programming interface, API

## Abstract

**Background:**

Pulse oximetry is a noninvasive method widely used in critical care and various clinical settings to monitor blood oxygen saturation. During the COVID-19 pandemic, its application for at-home oxygen saturation monitoring became prevalent. Further investigations found that pulse oximetry devices show decreased accuracy when used on individuals with darker skin tones. This study aimed to investigate the influence of X (previously known as Twitter) on the dissemination of information and the extent to which it raised health care sector awareness regarding racial disparities in pulse oximetry.

**Objective:**

This study aimed to explore the impact of social media, specifically X, on increasing awareness of racial disparities in the accuracy of pulse oximetry and to map this analysis against the evolution of published literature on this topic.

**Methods:**

We used social network analysis drawing upon Network Overview Discovery and Exploration for Excel Pro (NodeXL Pro; Social Media Research Foundation) to examine the impact of X conversations concerning pulse oximetry devices. Searches were conducted using the Twitter Academic Track application programming interface (as it was known then). These searches were performed each year (January to December) from 2012 to 2022 to cover 11 years with up to 52,052 users, generating 188,051 posts. We identified the nature of influencers in this field and monitored the temporal dissemination of information about social events and regulatory changes. Furthermore, our social media analysis was mapped against the evolution of published literature on this topic, which we located using PubMed.

**Results:**

Conversations on X increased health care awareness of racial bias in pulse oximetry. They also facilitated the rapid dissemination of information, attaining a substantial audience within a compressed time frame, which may have impacted regulatory action announced concerning the investigation of racial biases in pulse oximetry. This increased awareness led to a surge in scientific research on the subject, highlighting a growing recognition of the necessity to understand and address these disparities in medical technology and its usage.

**Conclusions:**

Social media platforms such as X enabled researchers, health experts, patients, and the public to rapidly share information, increasing awareness of potential racial bias. These platforms also helped connect individuals interested in these topics and facilitated discussions that spurred further research. Our research provides a basis for understanding the role of X and other social media platforms in spreading health-related information about potential biases in medical devices such as pulse oximeters.

## Introduction

Pulse oximetry is a noninvasive technique used extensively in critical care and other clinical environments to measure the oxygen saturation of blood. During the COVID-19 pandemic, the use of pulse oximetry for home oxygen saturation monitoring became widespread, allowing clinicians to track oxygen saturation levels, hence disease progression at home, with the goal of reducing avoidable emergency department visits [1]. Several applications of pulse oximetry, including self-monitoring at home, constant wireless monitoring in hospital facilities, and closed-loop oxygen delivery, were investigated during the pandemic [2]. With increasing monitoring, potential problems were noticed with pulse oximetry, such as accuracy concerns in skin colors and sources of error that could affect its use [3].

Subsequent investigations revealed that pulse oximetry measurement devices exhibit reduced accuracy when used on individuals with darker skin tones, a measurement issue that has been underappreciated for decades [4]. This phenomenon is attributed to the higher melanin content in individuals with dark skin, which leads to increased light absorption, impeding the accurate measurement of oxygenated blood levels by pulse oximeters [5]. Further investigations uncovered an adverse impact of pulse oximetry accuracy and evidence of statistical bias in pulse oximeter measurements, specifically in relation to race on patient care in black and colored patients, putting these patients at risk of hypoxemia and delayed escalation of care both in COVID-19– and non–COVID-19–related clinical scenarios [6-8].

Researchers called for more investigation into measures of bias, precision, and accuracy in the use of pulse oximetry to improve the interpretation of evolving research findings and facilitate the identification of potential solutions to these disparities [9]. Increasing awareness highlighted the racial bias in several medical devices, increased variation in monitoring data for Black patients, and its adverse impact on delivering evidence-based care, including pulse oximeter readings [10].

Health care professionals and the general public are now more aware of this issue due to campaign groups, advocacy, communities, and social media activism [3,11-13] and increasing awareness of disparities associated with pulse oximetry use in biomedical literature. With several hallmark social events around the time of the pandemic elaborating existing societal biases in health care that were disseminated globally through social media, we wondered about the role social media played in connecting social events to health care awareness on racial biases in a monitoring device such as pulse oximetry. We undertook this project to explore the impact of social media on increasing awareness of racial disparities in the accuracy of pulse oximetry and how information spreads among physicians. We used social network analysis to analyze the evolution of the conversations around pulse oximetry devices on X (previously known as Twitter) in relation to timelines of major social events raising health care disparities. Social network analysis has recently been used to explore and analyze the drivers of conspiracy theories [14,15].

We found that social media helped raise awareness of racial bias in pulse oximetry and should be considered a key communication channel alongside traditional methods such as medical publications and conferences. Our findings suggest that social media platforms have the potential to raise awareness about health inequities effectively and can be used to advocate for transformative regulatory action, adding to the growing body of evidence that social media can play a crucial role in advancing health equity. Our study is the first to analyze social media discussions on the specific topic of racial disparities in pulse oximetry accuracy.

## Methods

### Data Retrieval

The study adopted a retrospective review of social media activity, drawing upon data from X. Specifically, data were retrieved using the Academic Track application programming interface (API), as it was known then, using the search string: “pulseoximetry” OR “Pulse Oximetry” OR “Oximetry.” Searches were conducted using the X API each year (January to December) from 2012 to 2022 to cover an 11-year time period. This time period was selected as it generated the most sufficient data. We also examined literature indexed on PubMed between 1945 and 2020 and mapped it corresponding to 2012-2022. We captured data leading up to the end of 2022 when the US Food and Drug Administration (FDA) convened a group about ongoing concerns on the accuracy of pulse oximeters in individuals with darker skin pigmentations despite initial notification related to its use in the pandemic in February 2021.

### Data Analysis

**Figure 1:**
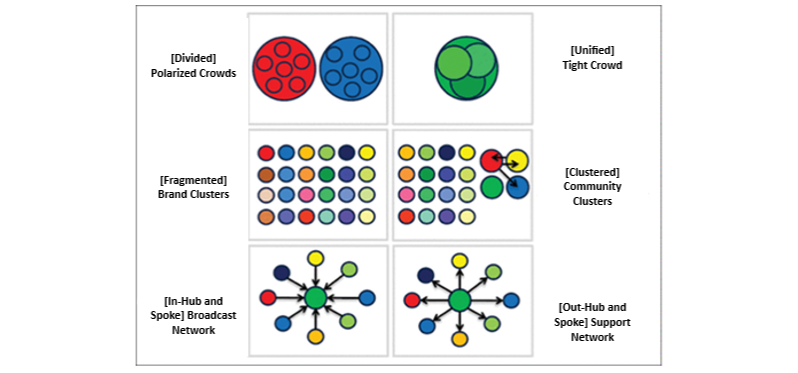
6 types of network structures that can emerge on X (formerly Twitter; sourced from Smith et al [18] and the Social Media Research Foundation).

Data were analyzed drawing upon Network Overview Discovery and Exploration for Excel (NodeXL, version 1.0.1.514; Social Media Research Foundation) to perform social network analysis and generate social network visuals. The Harel-Koren Fast Multiscale layout algorithm, which is useful for visualizing large undirected networks [14], was used to lay out the report. The nodes, entities like people or organizations between which connections are being explored, were clustered, drawing on the Clauset-Newman-Moore algorithm [16]. This gathered data specific to 2020 provided the most influential organizations and individual users on X participating in the discussion involving pulse oximetry. We also extracted their number of followers. NodeXL was also used to analyze the content of tweets to generate “word pairs,” which are able to show the 2 most frequently co-occurring words. Our study also drew upon the betweenness centrality algorithm [17]. Betweenness centrality is a method for determining the degree to which a node affects the information flow in a graph. It is frequently used to identify nodes that function as a connection between 2 sections of a graph. The algorithm calculates shortest paths between all pairs of nodes in a graph. We also categorized all posts based on the 6 types of X social network structures that can emerge [17], which are visually represented in Figure 1.

Polarized Crowds in X networks are formed by distinct, large groups discussing the same topic but with little to no interaction between them, often reflecting divergent views on contentious subjects like politics. Tight Crowds are formed around specialized topics or communities, such as professional interests, hobbies, or niche activities. In these networks, participants are highly interconnected, creating a dense web of interactions. Brand Clusters emerge when conversations on X revolve around specific brands or products without forming strong interconnections among the discussing participants. This results in many isolated individuals or small groups mentioning the brand but not engaging with one another, creating a scattered landscape of discussions. Community Clusters appear in conversations that encompass a broad topic with multiple subtopics or perspectives, forming several smaller, densely connected groups within the more considerable debate. The Broadcast Network structure is observed when a single, prominent account or a few accounts disseminate information to many followers, mirroring a hub-and-spoke model. Support Networks are identified by their hub-and-spoke configuration, where a central account, typically belonging to a company’s customer service department, interacts with numerous individual users.

Social media platforms have become central hubs for civil society, serving as venues for sharing information, engaging in public discourse, and hosting debates and conflicts [19]. As modern equivalents of the public square, the discussions on social media hold significant importance, comparable to documenting any substantial public gathering [18,20,21]. Creating network maps of these social media dialogues on platforms like X can shed light on the societal impact of social media. These maps resemble overhead snapshots of a crowd, revealing approximate population size and diversity.

Like topographic maps display mountain ranges’ peaks, network maps can reveal the most prominent points within a network landscape [18]. Specific individuals hold positions in these networks that are akin to strategically essential locations in physical terrain [18]. Using network metrics of “centrality,” it is possible to pinpoint key individuals who are pivotal in discussion networks, acting as leaders in the conversation. The content generated by these central figures tends to gain considerable popularity and is often widely shared across the network, highlighting their influential role in shaping social media discourse.

### Ethical Considerations

The study had ethical approval from Newcastle University (Reference 26055/2022), and we also took care to not draw attention to any individuals or accounts that were not already in the public domain. The majority of our analysis was conducted on an aggregate basis. During the editing process, we drew upon generative language models to improve readability and identify typographical errors of existing human-authored text with oversight from the authors, but these models were not used to generate any content of their own.

## Results

### Social Network and Interaction Analysis

**Figure 2:**
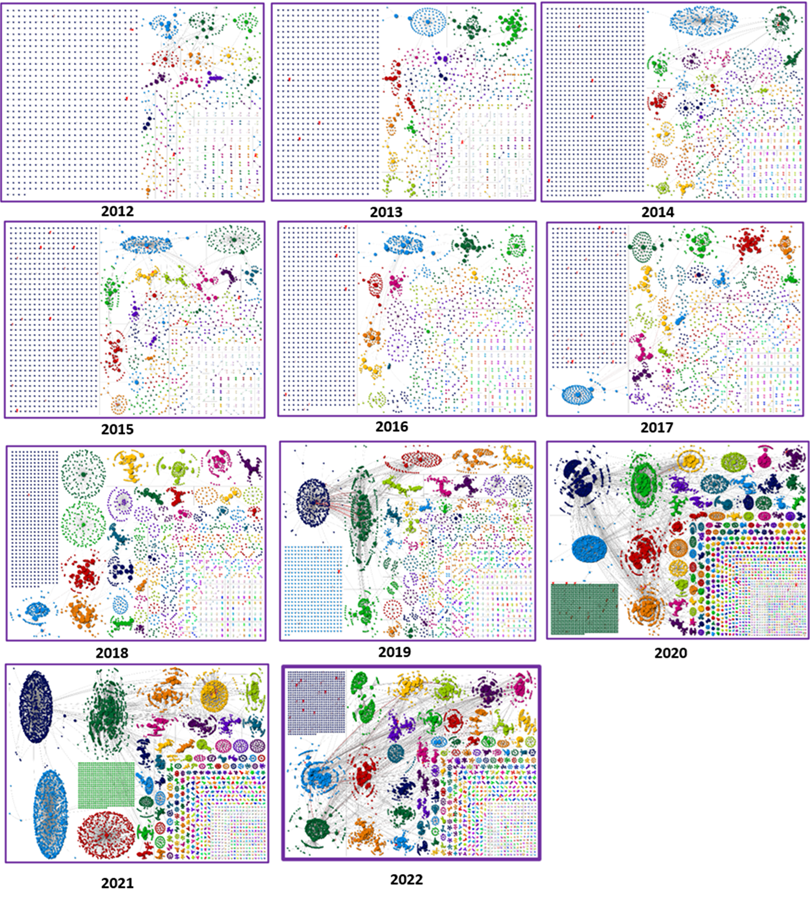
Social network analysis overview (2012 to 2022) of X posts and interactions (all forms of reposts and replies) on pulse oximetry.

The social network of pulse oximetry discussions on X from 2012 to 2022 is shown in Figure 2. We cut off in 2022 due to changes in the X (Twitter) API and are no longer able to access data through the Academic Track API. The social network analysis shows that the social network of pulse oximetry discussions on X has evolved significantly since 2019. In the early years (2012 to 2018), the largest group in each network consisted of a significant “isolates” group where users posted without being reposted and mentioning other users. Our analysis suggests that the topics were broad-ranging, with many “islands” of users conversing with each other. There was also very little cross-collaboration, suggesting that the networks lacked content and engagement. In 2019, there was a transformation as a “broadcast network” appeared for the first time as the largest group in the network. This change resulted from the discussion around pulse oximetry becoming more centralized, with a few key users influencing many others. There was also some evidence of cross-collaboration, suggesting that the networks were becoming more integrated. The most significant change occurred in 2020, when the network had been completely transformed, with many densely connected groups of users and several broadcast networks appearing. Users also appeared to be conversing across groups. Our results show that the discussion around pulse oximetry had become much more active and engaged, with various perspectives being represented. A similar pattern emerged in 2021 and 2022, with even larger individual groups and more evidence of cross-collaboration. This suggests that the social network of pulse oximetry discussions on X continued to evolve and mature. Overall, the social network analysis shows that the X pulse oximetry discussion has become more active, engaged, and integrated over time. This change in engagement is likely due to several factors, including the COVID-19 pandemic, which has increased public awareness of pulse oximetry.

**Figure 3:**
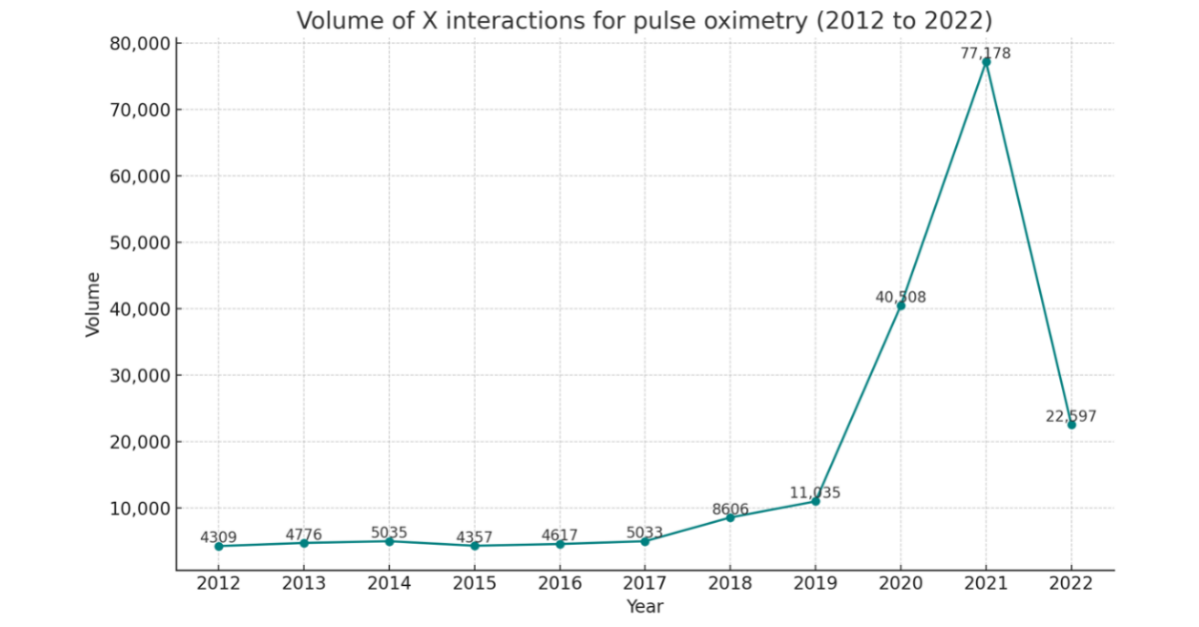
Interactions on the X platform.

**Figure 4:**
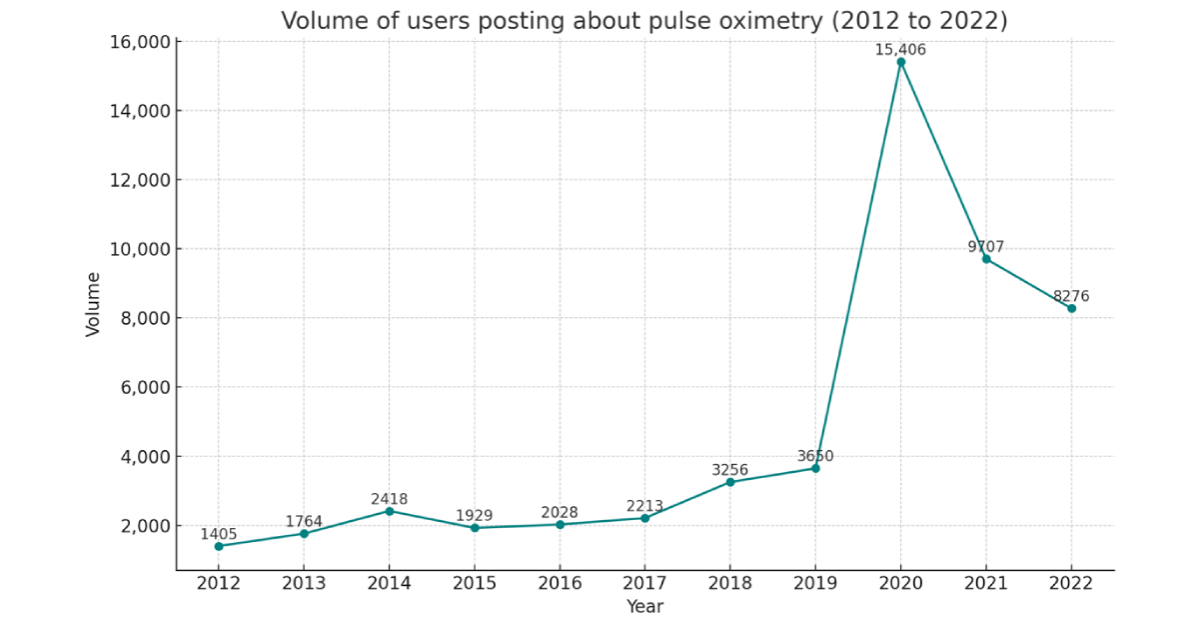
The volume of users posting.

Figure 3 shows the volume of X interactions, that is, all posts, all forms of reposts, and replies. Figure 4 shows the number of users within the networks over this time. For many years (2012 to 2017), activity remained similar, with a range of posts sent between 4309 and 5533. There was a slight increase in 2018 to 8606 posts and a more significant increase in 2019 to 11,035 posts, with a sudden jump in 2020 to 40,508 and a peak of 77,178 in 2021. While biomedical publications have traditionally had access through professional gatekeepers, we found that social media serves as a platform for individuals to widen existing information resources and connect with a broader network of health influencers [22,23]. In this case, specific health influencers and users on platforms like X played a role in highlighting the bias and discrimination of pulse oximetry technology. They emphasized the lack of formal health information available on the issue, leading people to turn to news articles or seek clarification through discussion topics and trends on X [24].

**Figure 5:**
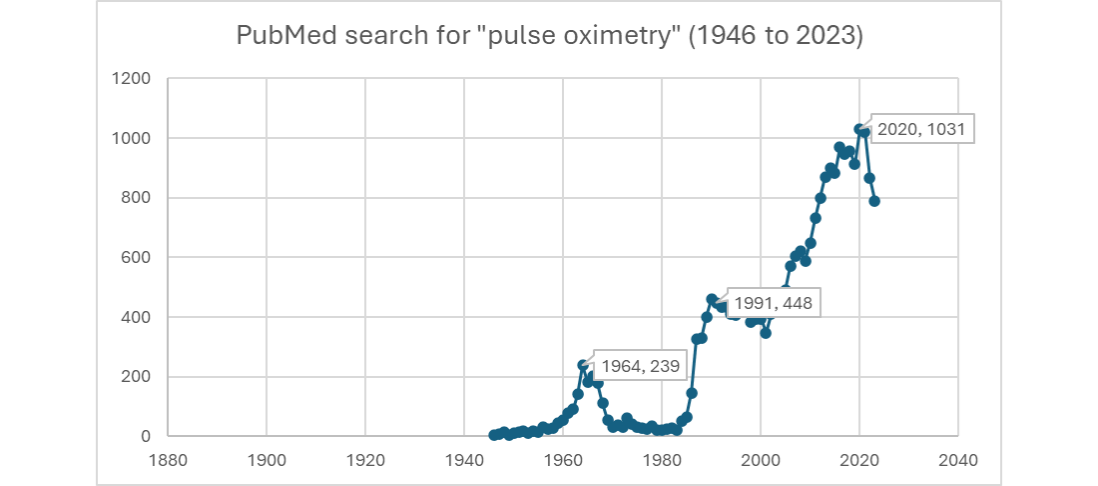
PubMed search for “pulse oximetry” (1946 to 2023).

Figure 5 provides an overview of all literature indexed in PubMed for “Pulse Oximetry” between 1946 and 2020. When searching specifically for the search string of “pulse oximetry racial bias,” PubMed highlights 2 papers published in 2020, a total of 10 papers in 2021, and 14 papers in 2022. These studies were widely shared and posted across X.

**Figure 6:**
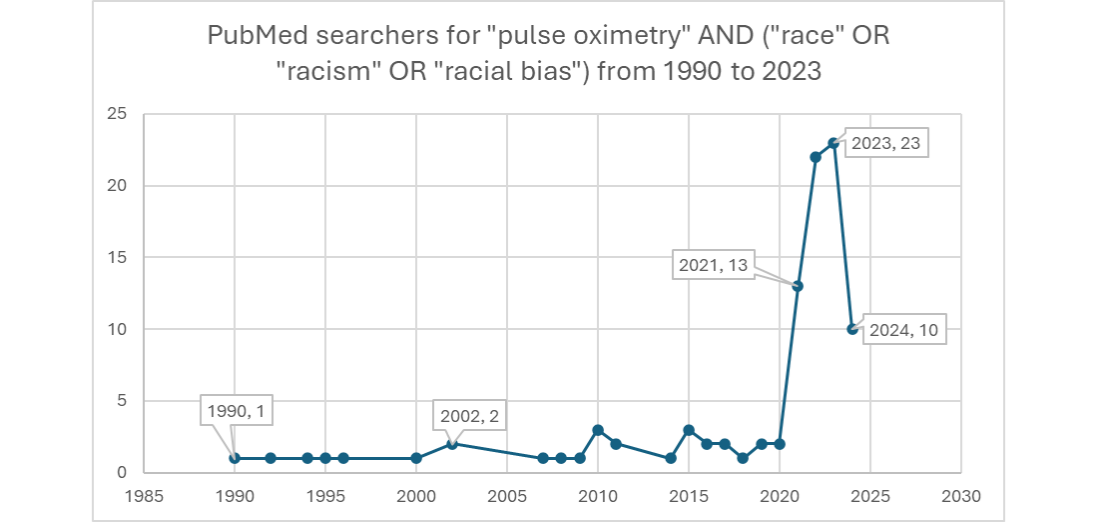
PubMed searches for pulse oximetry and race-related keywords (1990 to 2023).

**Figure 7:**
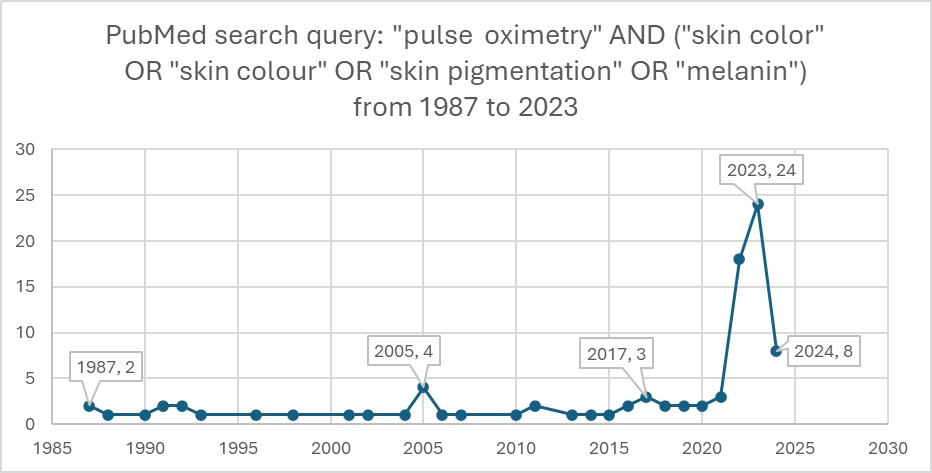
PubMed search for pulse oximetry and skin-related keywords (1987 to 2023).

The relationship between pulse oximetry readings and skin pigmentation has been known within the medical community for an extended period, as evidenced by research studies such as Adler et al [25], Bickler et al, [26], and Sjoding et al [27]. However, the significant implications of this correlation on racial disparities in health care outcomes remained largely unexplored and unaddressed until a surge in social media discussions around racial issues in 2019-2020. While a limited number of studies have explored knowledge sharing and data in this field, Jamali et al [28] shed light on a critical issue underscoring the racial disparity in oxygen saturation measurements by pulse oximetry. Sudat et al [29] and Shi et al [30] report similar findings and racial disparities at the height of the COVID-19 pandemic. This heightened awareness coincided with an increase in scientific research focused on the topic, indicating a newfound recognition of the need to understand and reconcile these disparities in medical technology and its application. This shift marks an essential step in addressing and rectifying biases in medical diagnostics that disproportionately affect people of color. Figures 6 and 7, created by searching PubMed for published papers, highlight how the increase of peer-reviewed publications on pulse oximetry coincides with the increased social media activity on X.

### Examining Key Sources and Information Shared in 2020

Several key web sources were frequently shared during discussions surrounding racial bias in pulse oximetry, shown in Table 1. The table includes details of the title, publisher, and number of shares.

**Table 1:** Overview of key sources, publisher, and number of shares (year 2020).

Source title	Publisher	Number of shares
Racial Bias in Pulse Oximetry Measurement	New England Journal of Medicine	327
The Infection That’s Silently Killing Coronavirus Patients	The New York Times	187
Takuo Aoyagi, an Inventor of the Pulse Oximeter, Dies at 84	The New York Times	57
Pulse oximetry to detect early deterioration of patients with COVID-19 in primary and community care settings	NHS^a^ England	50
COVID Oximetry @home	NHS England	48

^a^NHS: National Health Service.

The paper titled “Racial Bias in Pulse Oximetry Measurement” [27], published by the New England Journal of Medicine, emerged as a leading reference with 327 shares. The New York Times was also featured prominently in the discourse, with its articles covering various newsworthy aspects around pulse oximetry and other general COVID-19–related articles. In addition, content from the National Health Service, England, that provided general information on pulse oximetry during the COVID-19 pandemic, was frequently cited. The 3 sources were the most repeatedly shared sources during discussions surrounding racial bias in pulse oximetry. Due to the frequency of the sources, they were considered the most authoritative and informative sources on the topic. The sources were identified through a thorough search of web-based discussions and social media posts about racial bias in pulse oximetry. The frequency of sharing was used as a metric to determine which sources were the most prominent and influential.

The discussions surrounding racial bias in pulse oximetry revealed certain recurrent word pairs summarized in Table 2 that shed light on the focal points of the conversation. Table 2 provides insight into the top word pairs used together alongside their frequency.

**Table 2:** Overview of most frequently occurring word pairs in 2020.

Top word pairs	Occurrences
covid,19	2462
darker,skin	1499
oximetry,key	1494
management,covid	1479
clinical,management	1478
instrument,clinical	1477
inaccurate,darker	1477
key,instrument	1477

The pairing “COVID-19” with pulse oximetry with 2462 occurrences reflects the increasing interest in the device in monitoring patients with COVID-19. The word combination “darker skin” at 1499 instances highlights concerns about the potential inaccuracies of pulse oximetry readings in individuals with darker skin tones. This is further supported by the pair “inaccurate, darker” with 1477 mentions, which directly addresses the issue of instrument precision with darker skin. Other word pairs appear to highlight the importance and centrality of pulse oximetry in certain contexts, hinting at its foundational role in medical procedures or monitoring. Other discourse appeared to tie the tool’s clinical management capabilities, particularly in the management of patients with COVID-19. In the context of discussions on racial bias in pulse oximetry, several key influencers were identified based on betweenness centrality, which is summarized in Table 3. The table provides insight into the rank, name description, follower count, and location of the users who were most impactful.

**Table 3:** Overview of most influential users in 2020.

Influence rank	Name (on X)	Description	Number of followers	Location
1	Esther Choo, MD, MPH	Emergency physician and professor	201,890	United States
2	Matt Inada-Kim	National clinical director	2648	England
3	Ithan Peltan, MD, MSc	ICU^a^ and pulmonary physician and associate professor	513	United States
4	Utibe R. Essien, MD, MPH	Physician-scientist and assistant professor of medicine	25,782	United States
5	Faheem Younus, MD	clinician and a certified physician executive	496,647	United States

^a^ICU: intensive care unit.

The most influential user was Dr Esther Choo (MD, MPH), an emergency physician and professor from the United States. With an impressive following of 201,890, she stands out as a dominant voice on the subject. Following her is Matt Inada-Kim, the national clinical director from England. Although he has a notably smaller audience, boasting only 2648 followers, his influence in the discourse remains significant. The third influencer is Ithan Peltan (MD, MSc), an intensive care unit and pulmonary physician and associate professor from the United States. He engages with a community of 513 followers.

Another notable figure is Dr Utibe R. Essien (MD, MPH), also from the United States. As a physician-scientist and assistant professor of medicine, he has garnered attention from 25,782 followers. Finally, Dr Faheem Younus (MD), a clinician and certified physician executive from the United States, brings a substantial audience into the fold with 496,647 followers. The presence and reach of these influencers shaped the narrative and discussions.

## Discussion

### Principal Findings

The preliminary findings showed that the use of general keywords such as “pulse oximetry,” “Pulse Oximetry,” or “Oximetry” yielded a substantial cohort of individuals who expressed concerns or shared information regarding the racial bias inherent in the output of the pulse oximeter device. This observation implies an escalating level of interest on this subject on the X platform. Such conversations will typically occur in biomedical research, but these conversations were occurring on social media among the public, highlighting that people turned to social media. In addition, it was observed that several notable users used their position to increase public consciousness regarding potential concerns associated with pulse oximeters which highlights the influence of leaders through engagement in social media. The rapid and global dissemination of these posts highlights the influential capacity of X as a platform for expeditious information sharing. As modern equivalents of the public square, the discussions on social media hold significant importance [18,20,21]. Our study identified and categorized the primary clusters and users participating in this discourse. The tight shaping of the clusters demonstrates how users can form communities around topics of interest and that they can work together to help debunk issues and bias.

Whether the information on social media related to pulse oximetry and its racial bias was enough to change people’s behaviors in increasing conversation and advocacy around the topic is a complex question. Understanding the shape and types of influence among health networks on social media is essential for identifying the challenges of using social media for health communication. Abroms et al [31] acknowledge the potential of social media for improving public health while raising concerns about its negative impact on physical and mental well-being and its role in spreading misinformation. Relatedly, there is growing interest in the influence of professional health workers posting on social media. Engebretsen [32] emphasizes the importance of health practitioners maintaining a balance between establishing social proximity and maintaining professional detachment while disseminating health guidance on social media platforms such as Instagram (Meta). In addition, engaging responsible professional health care practitioners in disseminating valid and accurate information through social networks is essential to addressing the concerns raised by Abroms et al [31].

Here, the lack of formal access to health policy has forced health advocacy movements to advocate on a global and cross-platform level. Many social media users typically prefer 1 or 2 platforms, which means they may risk missing crucial health information. Vance et al [33] acknowledged the growing importance of social media platforms as sources of health information, especially among adolescents and young adults. However, they also emphasized the drawbacks of anonymous authorship and the dissemination of subjective viewpoints as objective facts. Pradekso et al’s [34] study highlights the importance of understanding the distinct health misinformation concerns linked with different types of social media.

Our findings highlight X’s potential as a platform for raising awareness for important health issues, even among those who may not be traditionally engaged with scientific research. We emphasize how health experts can use platforms like X to target specific communities, partnering with influential users and tailoring content to trending topics to appeal to different audiences.

The pandemic represented a significant inflection point for discussion in the public domain about racial bias in pulse oximetry. In response to the early stages of the pandemic, there was an increase in scientific papers concerning oximetry. The recent surge in research on racial bias in pulse oximetry stems from several factors. First, the COVID-19 pandemic reignited concerns about racial disparities in health care outcomes. This renewed focus exposed biases embedded in the design of medical devices [35], particularly in home-based products like pulse oximeters [36]. Second, the increased availability of data on pulse oximetry readings and patient outcomes provided researchers with real-world evidence of these biases [30,37]. Finally, heightened public and scientific interests in the topic attracted additional funding for health research efforts [38]. With the increased number of studies on racial bias after 2020, we can speculate that the rigor of the studies also improved due to the availability of data, the development of new analytical methods, and the growing number of researchers working in this area.

Social media platforms like X allowed researchers, health experts, patients, and the general public to share information quickly and widely, raising awareness about the potential for racial bias, finding other people interested in the topics and generating discussions for further research. Our study, therefore, has some limitations. First, selection bias is limited to a single sample of posts on one platform. Further research encompassing different platforms and related terms is needed to confirm our findings. Second, we did not collect demographic data on the users in our sample, so we cannot say who is most likely to be exposed to information about racial bias in pulse oximeters. Third, we did not perform a sentiment analysis or qualitative analysis of the posts to show advocacy-related follow-up, so we cannot say how users respond to this information. Fourth, we could not objectively report how these could have tied into increasing scientific discourse on the topic or heightened the need for action for regulatory authorities such as the FDA. Fifth, a deeper understanding of the context in which race and oximetry are being discussed is not there, as our study looks at activity generated by posts. However, our future work aims to better understand the context of the discussions. Finally, the reliance on social media data presents another limitation. Platforms such as X, while offering rapid access to health information, have their own limitations. Concerns exist regarding potential biases within platform algorithms, as evidenced by Savolainen’s (27) work on algorithmic governance and user struggles with platform expectations. This highlights users’ potential difficulty in accessing valid health information on social media.

Notwithstanding these constraints, our research offers a crucial foundation for understanding the function of X and other social media platforms in disseminating health-related information about potential biases in health devices such as pulse oximeters. Social events are invariably discussed in social media–fueled scientific discourse on a topic known to the scientific community for decades but only researched in relation to health care disparities after social media raised awareness. Future research should aim to expand upon our study by using more comprehensive and diverse sampling techniques, gathering user demographic information, and using qualitative analysis of posts to gain a deeper understanding of user reactions. Furthermore, research by Philip et al [9] during the pandemic emphasized the need for a critical re-evaluation of pulse oximetry technology. Their work highlighted concerns about racial bias in pulse oximetry readings and proposed modifications to account for patient skin color variations. In response to data bias, Ferrari et al [39] advocated for implementing multi-wavelength pulse oximeters that incorporate melanin absorption correction to enhance accuracy, particularly in the context of the COVID-19 pandemic. These studies parallel the concerns we observed in social media discussions, further highlighting the need for continued research and technological improvements.

### Conclusion

Our analysis of social media discussions revealed a significant increase in public interest surrounding racial disparities in pulse oximetry readings. These findings highlight the need for increased public awareness and education about pulse oximetry limitations. In addition, our study provides valuable insights for future research efforts focused on improving pulse oximetry technology and addressing identified biases. Ultimately, ongoing collaboration between health care professionals and the public is crucial for ensuring the fair and accurate use of pulse oximetry for all patients. Furthermore, this study demonstrates the potential of social media platforms, such as X, as a space for disseminating academic research about racial bias in pulse oximetry. We observed key influential users leveraging their platforms to raise awareness of these issues and rapidly disseminate early scientific findings. These results offer valuable insights for raising awareness of critical health issues and understanding health information propagation on social media.

Our analysis suggests that social media can significantly enhance health information sharing and advocacy strategies, particularly for nonhealth professionals. This study highlights the role of key influencers and network links on social media in shaping health information dissemination. These influencers and network connections crucially impact how health organizations use social media and how individuals engage in health social movements like advocacy. While traditional, offline health information channels aimed at patient education remain important, our findings suggest that influence in the health sphere is increasingly intertwined with, or even dependent upon, the presence of key actors on digital platforms.

## Abbreviations

API: application programming interface
FDA: US Food and Drug Administration
NodeXL: Network Overview Discovery and Exploration for Excel
